# Assessment of Ocular Surface Pathogen Susceptibility to Ultraviolet‐C (UVC) Light Treatment With a Novel Prototype Device—An In Vitro Study

**DOI:** 10.1111/vop.70214

**Published:** 2026-06-19

**Authors:** Oliver Joe Williams, Charlotte Dawson, Maria‐Christine Fischer, Siân‐Marie Frosini

**Affiliations:** ^1^ Department of Clinical Science and Services, The Royal Veterinary College Queen Mother Hospital for Animals Hertfordshire UK; ^2^ Department of Pathobiology and Population Sciences The Royal Veterinary College Brookmans Park UK

**Keywords:** adjunctive therapy, bacterial keratitis, UVC light therapy

## Abstract

**Purpose:**

Assess in vitro efficacy of a device emitting 265 nm UVC light against bacteria isolated from veterinary infectious keratitis.

**Methods:**

Twenty‐seven clinically‐derived bacterial isolates: *Staphylococcus pseudintermedius* (*n* = 10; including *n* = 2 methicillin‐resistant *Staphylococcus pseudintermedius* [MRSP]), *Staphylococcus aureus* (*n* = 1), *Streptococcus canis* (*n* = 4), *Escherichia coli* (*n* = 4, including *n* = 2 multidrug‐resistant isolates), *Pseudomonas aeruginosa* (*n* = 7) and *Serratia marcescens* . (*n* = 1), and three type culture strains (*n* = 1 *E. coli* , *S. aureus,* *S. pseudintermedius* ) were lawn cultured. Prototype UVC device (2.50 mW/cm^2^ intensity, 23 mm diameter beam) provided triplicate exposures at 1, 2, 3, and 5 s, and plates incubated (37°C, 16–20 h). All experiments were performed in duplicate (*n* = 6 treatment zones per timepoint, per isolate). Absence of growth in all exposed areas at any time demonstrated complete UVC inhibition; presence of growth was categorized as < 10 or ≥ 10 colonies.

**Results:**

UVC inhibition was complete in 11/30 isolates at ≤ 5 s exposure. Partial efficacy was seen in 18/19 remaining isolates at 5 s; ≥ 33% zones demonstrated absence of bacterial growth. Efficacy against MDR‐MRSP and MDR‐ *E. coli* was comparable to susceptible counterparts. All *P. aeruginosa* were completely inhibited at ≤ 5 s; the *S. marcescens* isolate was least susceptible with ≥ 10 bacterial colonies within 50% zones after 5 s.

**Conclusion:**

Five seconds of UVC exposure is sufficient to markedly reduce growth of most bacterial species, including MDR‐isolates and completely inhibit *P. aeruginosa* in vitro. These findings support further controlled in vivo safety and efficacy studies of UVC as an adjunct to topical antibiotics in companion animal infectious keratitis.

## 1 Introduction

Ulcerative keratitis is a common presentation of ophthalmic disease in veterinary species in both general practice and in referral settings [1, 2]. The healthy corneal epithelium is highly protective against bacterial invasion and furthermore intrinsic anti‐bacterial mechanisms exist within the pre‐corneal tear film such as lysozyme, lactoferrin, and immunoglobulins [3]. Despite these mechanisms, the ocular surface hosts a microbiological commensal flora, which has no clinical implication in healthy eyes. However, it has been demonstrated that the ocular microbiota may be altered in the presence of ocular surface disease, such as keratoconjunctivitis sicca, causing dysbiosis and allowing for opportunistic invasion [4, 5], as well as promoting a more pathogenic flora such as coagulase‐positive *Staphylococcus aureus* [6]. Many of these ocular surface diseases also predispose to ulcerative keratitis, which could explain the high incidence of infected ulcerative keratitis when ocular co‐morbidities exist [7, 8].

Ulcerative keratitis may become complicated by bacterial invasion into the exposed stroma. Resulting collagenolysis is driven by proteases which may be bacterially derived such as in *Pseudomonas* spp., or host derived secondary to an influx of leukocytes and cytokines [9, 10]. One of the mainstays of treatment in any form of ulcerative keratitis is therefore administration of topical antibiotics, either in a prophylactic fashion or against active infection. Culture results support selection of appropriate antibiotics, but there is an unavoidable delay in transport of samples and following 24–48 h time to culture pathogens. The necessity for prompt treatment forces selection of an empirical antimicrobial whilst pending confirmation of predicted efficacy. Increasing antimicrobial resistance has been widely described, particularly to first line treatments and increasingly to fluoroquinolones [11, 12, 13], although the relevance of susceptibility reports when considering a topical therapy is questionable. There is an increasing drive to reduce antimicrobial selection pressure, and to mitigate the spread of antimicrobial resistance, through investigating non‐traditional antimicrobial therapeutic approaches. To date a number of alternatives have been investigated as sole and adjunctive therapies such as topical povidone iodine, ozone‐based eye drops and photoactivated chromophore for keratitis‐corneal cross‐linking (PACK‐CXL) [14, 15, 16, 17].

Ultraviolet (UV) light has been reported to have bactericidal activity since the early 19th century [18]. UV mediated DNA damage occurs through photodimerization of two adjacent pyrimidine bases, forming lethal DNA “lesions” such as cyclobutane pyrimidine dimers (CPDs), pyrimidine 6–4 pyrimidone photoproducts (6–4PPs) [19]. When UV is separated into types A, B, and C, variability of efficacy between these groups exists [20]. Of the UV spectrum, UVC (100–280 nm) is the most germicidal; however, it is completely screened from the earth's surface by ozone [21]. UVC gained the name Germicidal UV, with its effects being utilized for water, milk, and air disinfection during the last 100 years, regaining significant interest during the COVID‐19 pandemic [22, 23, 24, 25].

UVC has gained interest for its therapeutic potential in sterilization of superficial wound infections and particularly, over the last two decades, for its application in treating infectious keratitis [26, 27]. The extremely limited tissue penetration and an apparent biophysical sweet spot of radiation energy at which bacterial sterilization occurs with minimal effect to host DNA make UVC an ideal treatment option [28, 29]. Furthermore, any host DNA lesions that occur are readily reversed by intrinsic DNA repair mechanisms [19, 30]. A device utilizing 265 nm UVC light emitting diode (LED) to produce a 4.5 mm spot for the treatment of microbial keratitis has been extensively tested for safety and efficacy via in vitro, ex vivo, and in vivo murine model studies [30, 31, 32]. This has subsequently been developed to produce a device emitting a 23 mm diameter beam, potentially of utility in the treatment of microbial keratitis in veterinary species. In the last 2 years, four veterinary studies have been published by a group from Iowa State University, investigating the utility of a modified, commercially available handheld UVC device (275 nm) for treating canine bacterial infectious keratitis, bovine *Moraxella bovis* , and equine keratocmycosis in vitro and ex vivo [33, 34, 35, 36].

The present study aimed to assess the in vitro efficacy of UVC using a prototype device emitting a 23 mm diameter beam with a wavelength of 265 nm (Photon Therapeutics Ltd), against bacteria isolated from clinical cases of infectious keratitis in companion animals and multidrug‐resistant bacteria.

## 2 Methods and Materials

Twenty‐five bacterial isolates were sourced from the Royal Veterinary College's diagnostic microbiology laboratory, collected from clinical submissions undergoing bacteriological culture and susceptibility testing with identification via MALDI‐TOF (VITEK‐MS, BioMerieux, Basingstoke, UK) and susceptibility testing through broth microdilution (VITEK‐2, BioMerieux). Isolates comprised 11 isolates stored retrospectively from another ongoing infectious keratitis study and 14 isolates stored prospectively from August 2023 to December 2023 following the inception of the study.

Furthermore, two MDR, methicillin‐resistant *S. pseudintermedius* (MRSP) originating from canine skin and ear infections as part of a previous study, representing two different sequence types and characterized genetically (23 929, 1726) [37], and three type culture strains were tested (ATCC 25922 *E. coli*, ATCC 25923 *S. aureus*, LMG 22219 *S. pseudintermedius* ).

Each isolate (*n* = 30) was sub‐cultured onto 5% sheep's blood agar (CM0055B, Oxoid, Basingstoke, UK; SB054, TCS Biosciences, Buckingham, UK) at 37°C for 24 h to ensure purity prior to UV‐exposure. For UVC exposure testing, Mueller‐Hinton agar (CM0337B, Oxoid) was used for all isolates except the four *S. canis* , for which Iso‐Sensitest agar with lysed horse blood (PB0145A, Oxoid) was used due to their fastidious nature. Preparations of bacterial lawns were performed in accordance with The European Committee on Antimicrobial Susceptibility Testing (EUCAST) antimicrobial susceptibility testing guidelines. A 0.5 McFarland standard, equivalent to (1.5 × 10^8^ colony forming units (CFU)/mL) bacterial suspension in sterile distilled water, confirmed by Densichek (BioMerieux) was made, and 100 μL inoculated onto each of four separate plates and spread to ensure complete coverage across the surface of the agar.

**FIGURE 1:**
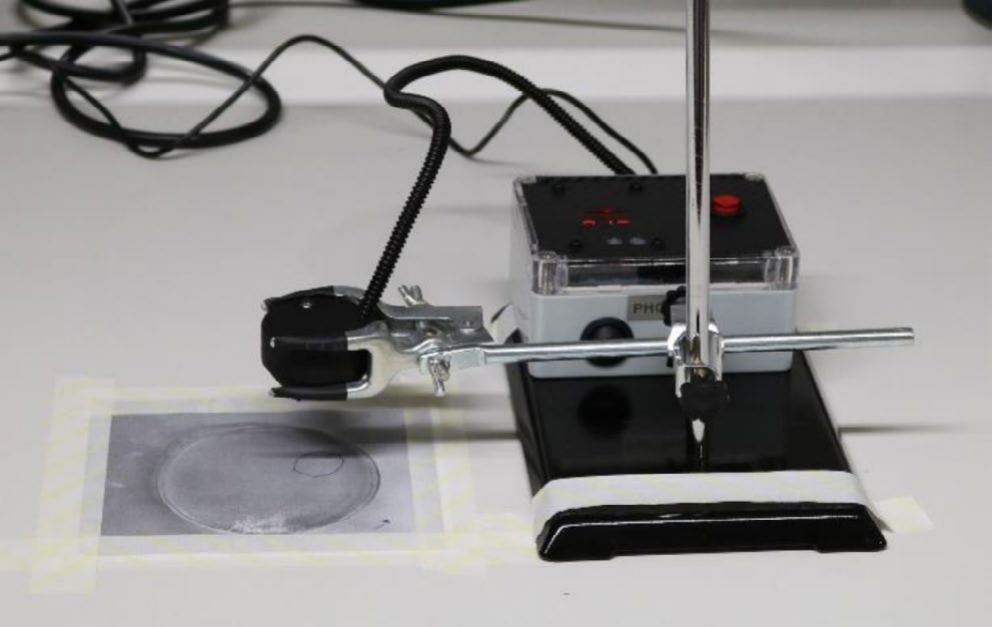
The prototype UVC device and laboratory setup.

A prototype UVC device was loaned from Photon Therapeutics Ltd. (London, United Kingdon). The optical head contained an LED that produced UVC at a peak wavelength of 265 nm with a 23 mm diameter circular beam. This was fixed at a working distance of 30 mm above the surface of the plates (Figure 1) using a laboratory stand and clamp. At this distance the resulting intensity was reported as 2.50 mW/cm^2^. Triplicate exposures of 1, 2, 3, and 5 s were performed across three separate areas of each of the four plates per isolate. The equating energy doses (energy dose = intensity × time) are calculated to be 2.50, 4.99, 7.48, and 12.47 mJ/cm^2^ respectively. The plates were then inverted and incubated aerobically at 37°C for 16–20 h. The exposed zones were subsequently assessed for bacterial growth inhibition and recorded as no bacterial growth or bacterial growth. If growth was present, this was recorded through colony counting and grouped for analysis as < 10 or ≥ 10 individual colonies. The entire process was performed in duplicate across two separate occasions, resulting in six UVC exposed zones of bacterial lawn to assess for growth for each time point and each isolate.

Complete inhibition was defined as no bacterial growth in all UVC exposed zones across both replicates (*n* = 6) for an isolate at a given exposure time. The term partial efficacy was used when bacterial colonies were present within one or more UVC exposed zones; however, the number of individual bacterial colonies was less than 10.

## 3 Results

The 25 clinically derived isolates were comprised of coagulase‐positive staphylococci (*n* = 9) (*n* = 7 *Staphylococcus pseudintermedius, n* = 1 multidrug‐resistant [MDR] *S. pseudintermedius* , *n* = 1 *Staphylococcus aureus* ), *Streptococcus canis* (*n* = 4), *Escherichia coli* (*n* = 4, including *n* = 2 MDR isolates), *Pseudomonas aeruginosa* (*n* = 7) and *Serratia marcescens* (*n* = 1 MDR isolate). MDR was defined as resistance to three or more classes of antimicrobials [38], not including predictable/intrinsic resistances; where not stated as MDR, isolates were susceptible to most antimicrobials reported by the laboratory (File S1). Twenty‐three isolates were from cases of infectious keratitis and sample location was recorded for 22 of these. Sample location was recorded as corneal (*n* = 18), conjunctival (*n* = 3) or the eyelid (*n* = 1). The three isolates of conjunctival origin also cultured positive for the same pathogen on concurrent corneal sampling and therefore the isolate was representative of the infectious keratitis. The isolate collected from the eyelid (*S. pseudintermedius 3*) was assumed to be representative of the infectious keratitis due to entropion causing corneal irritation; however, the cornea itself was not sampled in this case. Two isolates (*n* = 1 MDR‐*E. coli, n* = 1 *P. aeruginosa* ) were cultured from a bronchoalveolar lavage and a surgically excised subcutaneous abscess, respectively. They are pathogens known to cause infectious keratitis and were included to assess MDR isolates and to increase biological variation in pathogens. Of the isolates with recorded species origin, 23 were canine‐derived, and one was feline‐derived.

Complete inhibition was observed for 11 of 30 isolates at ≤ 5 s exposure; of those, four isolates were completely inhibited by 2 s, three by 3 s, and four by 5 s (Table 1). Partial efficacy was seen in 18 of the remaining 19 isolates (Table 1). The remaining *S. marcescens* isolate had growth > 10 colonies in half of the exposed zones at 5 s; however, there were two zones of no growth and one zone with growth of less than 10 individual colonies.

**TABLE 1:** Assessment of growth of *n* = 30 bacterial isolates following exposure to 2.50 mW/cm^2^ UVC for 1, 2, 3 and 5 s (*n* = 6 replicates of exposed areas).

Bacterial species	Isolate code	Multidrug‐Resistant (yes/no)^a^	% exposed zones with no bacterial growth at time point				% exposed zones with < 10 individual colonies or no bacterial growth at 5 s
			1 s	2 s	3 s	5 s	
*Staphylococcus pseudintermedius*	SP1	YES	0%	0%	17%	67%	100%
	SP2	No	0%	0%	50%	50%	100%
	SP3	No	0%	0%	0%	50%	100%
	SP4	No	0%	0%	67%	67%	100%
	SP6	No	0%	0%	0%	67%	100%
	SP7	No	0%	0%	67%	67%	100%
	SP8	No	0%	0%	33%	33%	100%
	SP9	No	0%	0%	17%	83%	100%
*Staphylococcus aureus*	SA5	No	0%	33%	50%	67%	100%
Methicillin‐resistant *S. pseudintermedius*	MRSP1	YES	0%	0%	20%	60%	100%
	MRSP2	YES	0%	0%	50%	50%	100%
*Streptococcus canis*	SC1	No	0%	17%	17%	67%	100%
	SC2	No	0%	0%	0%	0%	100%
	SC3	No	0%	33%	33%	50%	100%
	SC4	No	0%	0%	17%	50%	100%
*Escherichia coli*	EC3	YES	0%	0%	0%	100%	100%
	EC4	YES	0%	17%	100%	100%	100%
	EC6	No	0%	0%	50%	67%	100%
	EC8	No	0%	33%	33%	83%	100%
*Serratia marcescens*	SM	YES	0%	0%	0%	33%	50%
*Pseudomonas aeruginosa*	PA1	No	0%	100%	100%	100%	100%
	PA2	No	0%	100%	100%	100%	100%
	PA3	No	50%	67%	100%	100%	100%
	PA4	No	0%	100%	100%	100%	100%
	PA5	No	0%	17%	67%	100%	100%
	PA6	No	0%	17%	50%	100%	100%
	PA7	No	33%	100%	100%	100%	100%
*E. coli* ATCC 25922	ATCC 25922	No	0%	0%	33%	50%	100%
*S. aureus* ATCC 25923	ATCC 25923	No	0%	0%	100%	100%	100%
*S. pseudintermedius* LMG 22219	LMG 22219	No	0%	0%	50%	100%	100%

*Note:* Green shaded cells depict 100% of exposed zones depicting complete efficacy whereas the orange cells depict greater than 0% but less than 100%, therefore partial efficacy.

^a^ Multidrug‐resistance defined as resistance to ≥ 1 antimicrobial in ≥ 3 classes.

**FIGURE 2:**
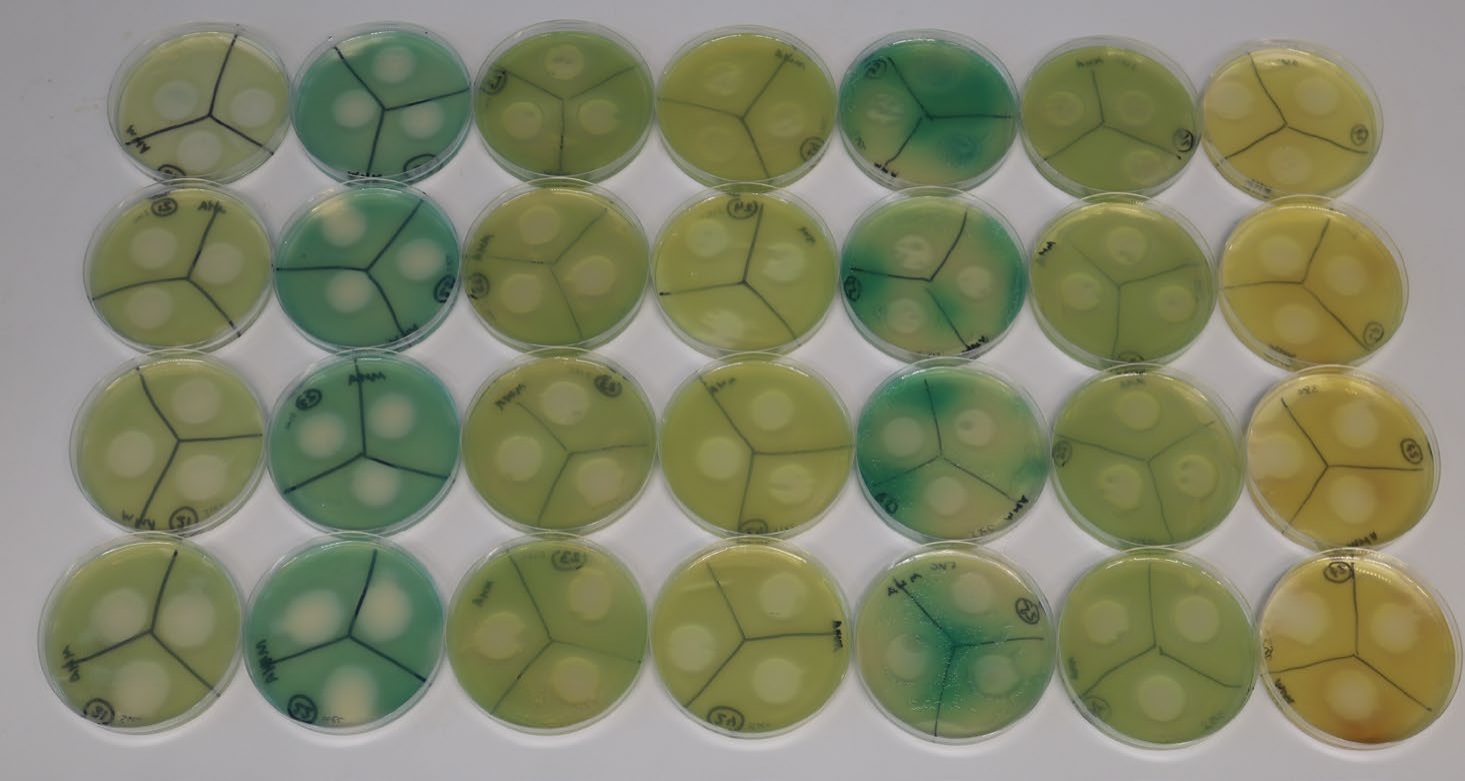
*P. aeruginosa* plates following exposure. Left to right: Isolates 1 to 7. Top to bottom: Exposure times from 1 to 5 s. Complete growth inhibition of all isolates is seen by 5 s UVC exposure.

Complete inhibition was observed for all tested *P. aeruginosa* isolates, (*n* = 7) ≤ 5 s, with four of these being completely inhibited by 2 s (Figure 2). *E. coli* isolates showed a mixture of inhibitory effects, although *n* = 2 MDR‐ *E. coli* were completely inhibited by 3 and 5 s, respectively. UVC efficacy appeared comparable between the MDR‐*S. pseudintermedius*, MDR‐MRSP and susceptible *S. pseudintermedius* isolates tested, although partial efficacy was observed for all isolates except for the type culture (LMG 22219 *S. pseudintermedius* ) which displayed complete inhibition.

## 4 Discussion

The in vitro results from this study demonstrate that 265 nm UVC irradiation has a dose dependent effect and inhibits bacterial growth to increasing degrees regardless of species and antimicrobial resistance profile. Minimum effective exposure times could be established for 37% (11/30) of isolates tested in this study however strict criteria were utilized, with the growth of even a single colony within a single exposed zone (out of six) constituting treatment failure. This extremely stringent definition was deliberately elected in order to replicate previous studies as well as minimize the risk of over‐estimating efficacy and err on the side of under‐estimating biological activity. Of the remaining isolates, all but one isolate demonstrated partial efficacy indicating marked inhibitory effect on the growth of the bacteria following UVC exposure. Although the 10‐colony threshold is arbitrary, this semi‐quantitative threshold represents an estimated log5 reduction in bacterial burden. Furthermore, this classification mimics traditional antimicrobial disc diffusion interpretation of clear inhibition, occasional microcolonies or substantial growth. This nuanced classification provides likely relevance to clinical use of UVC as an adjunct therapy to reduce bacterial load in an infection. A substantial and rapid reduction in bacterial burden would support the intrinsic antimicrobial properties of the tear film, the host immune system and/or topical antibiotics. Interestingly, one isolate in the partial efficacy group, *S.canis* 2, did not display any zones of growth inhibition across all six zones exposed to UVC at 5 s. It is unclear why a reduced effect of UVC was observed for this isolate however it likely relates to the Gram positive cell structure and a protective role of the thick peptidoglycan cell wall against UVC [39]. Based on this data, complete sterilization of *S. canis* species with UVC alone may require longer durations of exposure beyond 5 s, higher intensities or repeated exposures after set time periods such as has been demonstrated previously [33].

UVC failed to completely inhibit a single isolate, *Serratia marcescens* , with growth of 10 or more colonies in half of the exposed zones at 5 s. Although this pathogen is less commonly isolated in ulcerative keratitis in veterinary species, association with contact lens use in human ophthalmology has been demonstrated [40]. It would be important to test a larger number of *Serratia* spp. isolates to confirm whether the increased tolerance is a species‐wide property. It is of note that some strains of this Gram‐negative rod possess the ability to produce a UV‐protective pigment called prodigiosin [41, 42], and although no pigmentation was observed grossly when culturing the isolate, further investigation with a larger number of isolates with phenotypic confirmation is needed.

The results of the present study suggest that the maximal energy dose assessed (12.47 mJ/cm^2^) was not sufficient to completely inhibit all isolates. This would insinuate a lesser efficacy than has been observed previously at the same UVC wavelength tested [31, 32] and more recently when investigating the use of a longer wavelength, 275 nm, delivered through a modified commercially available portable UVC device at 10 mm distance [33]. Interestingly, however, the UVC inhibition results of the present study become comparable to those of Turicea et al. 2024 as the working distance is increased to 15 mm. At this distance, a 15 s dose resulted in an energy dose of 15 mJ/cm^2^ and only produced complete inhibition in 3 of 10 isolates. A second treatment was required after 4 h to completely inhibit the remaining isolates.

Another key difference that must be noted is the size of the exposed areas. The previous studies utilized a 4.5 mm UVC treatment spot [30, 31, 32], and 3 mm collimation resulting in a UVC treatment spot of 7 or 8 mm diameter depending on working distance [33], compared to 23 mm as used in the present study. This significantly larger exposed zone incorporates a significantly higher number of CFU within it. Furthermore, the concentration of bacterial inoculum in the current study was chosen to align with antimicrobial susceptibility testing criteria as set by EUCAST. This is a much higher concentration inoculum, around 150‐fold higher, than utilized in previous studies (Dean al. 2011 and Marasini et al. 2022). When combining the increased CFU/mL and the increased exposed area, it represents a nearly 4000‐fold increase in the number of CFU within the respective exposed zones. Considering that bacterial burdens of 10^8^–10^9^ cfu/mL have been described in human infections [43], it is reasonable to consider that testing novel antimicrobial devices on populations of bacteria comparable to those used in conventional susceptibility testing is important to allow extrapolation of results to support prediction of clinical efficacy. The quantitation of bacterial burden in infectious keratitis in veterinary species has not been described to date.

Although this larger treatment zone allows concurrent treatment of the whole cornea in canine and feline species, there is no option to collimate the beam to a smaller size to tailor to individual cases. This results in UVC exposure of healthy adjacent corneal tissue and importantly the limbus with residing stem cells. Studies assessing both an in vivo rat model and porcine ex vivo models have demonstrated that UVC penetration and CPDs were present at the level of the limbal stem cells in rats but are spared by UVC exposure due to the superficial penetration in porcine corneas. Further safety assessment is necessary to ensure this correlates for domestic species [44, 45].

Another possible limitation was that plates were incubated after one to 3 h at room temperature on the laboratory bench. Whilst all plates were handled under consistent ambient laboratory lighting conditions, that was the same across all replicates, light conditions were not formally controlled, representing a study limitation. This window of time prior to incubation may have provided the bacteria the opportunity to undergo photoreactivation and thereby allow DNA repair. This is a process whereby photolyase binds directly to the UV induced CPDs and 6–4PPs. Photolyase then absorbs light from the visible, particularly blue, spectrum to cleave the thymine dimer back to adjacent thymine monomers [46, 47]. This counteraction of the UVC effect could not be demonstrated with 12 h of 254 lx artificial light after an initial 6 or 12 h in the dark when assessed for *Moraxella bovis* in vitro [35], however, further studies are required to investigate this further with canine isolates and variable periods of differing lux light exposure following treatment.

The results of this study demonstrate that UVC may be a particularly useful adjunctive treatment in infected ulcerative keratitis in companion animals, alongside topical antimicrobial therapy. This aligns with the need for Antimicrobial Stewardship interventions and reduction of antimicrobial use in an era of increasing antimicrobial resistance. In particular, it is reassuring to see comparable efficacy of UVC against MDR‐pathogens and those that are considered complex for antimicrobial selection due to intrinsic resistances; the demonstrable susceptibility in particular of *P. aeruginosa* isolates to UVC in this study is very positive. All isolates tested displayed complete growth inhibition by 5 s exposure, with the majority of these by only 2 s of exposure, corresponding to energy doses of 12.47 mJ/cm^2^ and 4.99 mJ/cm^2^ respectively. This is further supportive of the increased in vitro UVC susceptibility of *P. aeruginosa* from canine infectious keratitis [33], and in a murine in vivo model [31]. The reported rates of *P. aeruginosa* infected ulcerative keratitis in dogs ranges from 7.6% to 41% [11, 48], and significant associations have been reported including melting at time of sampling, more severe ulceration and resultant globe loss [12, 49, 50]. Furthermore, *P. aeruginosa* possesses intrinsic resistance to the most common first line topical antimicrobials utilized in ophthalmology for companion animals in the United Kingdom, chloramphenicol and fusidic acid. These results support future assessment of adjunctive UVC therapy alongside topical antimicrobials with the hope that it may aid rapid control of infection, halt progression of disease, and improve outcomes in infected ulcerative keratitis.

One limitation of this study was that longer treatment durations and therefore higher energy doses were not assessed. The decision to maximize exposure at 5 s was a limitation of the prototype device; longer durations of treatment may have caused damage to the LED within the optical head. Furthermore, 5 s of treatment was predicted to be sufficient based on equivalent energy doses from prior efficacy studies with the preceding prototype [31, 32]. Finally, it was also justified on the basis of clinical applicability; it was considered that the majority of conscious veterinary patients would be tolerant of a continuous 5 s treatment without movement or blinking. However, since conducting this study, the therapeutic device (PhotonUVC Vet, Photon Therapeutics Ltd) has become commercially available for clinical treatment in veterinary species. The therapeutic device provides a higher intensity UVC dose at the same working distance and therefore a greater energy dose over the same duration of time (5 s). To deliver an equivalent dose with the prototype device tested in this study, a 7 s exposure would have been required. If extrapolating from the data generated with 1–5 s of exposure in the present study, it could be predicted that a 7 s exposure would demonstrate greater antimicrobial efficacy, relating to a higher energy dose delivered. This extrapolation would, however, require confirmation with further in vitro testing.

## 5 Conclusion

This study demonstrates an increasing, dose dependent in vitro efficacy of UVC for bacterial growth inhibition against clinical isolates when using a prototype device emitting a 23 mm diameter beam with a wavelength of 265 nm. Whilst complete inhibition was not achieved for all isolates, good antimicrobial efficacy was seen for the majority of isolates. In particular, bacteria that present increased challenges for conventional antibiotics, such as MDR‐MRSP and *P. aeruginosa* isolates exhibited vast reduction in bacterial survival after 5 s of exposure. This demonstrates a clear potential for UVC as an adjunctive therapy, with the aim to reduce duration of topical antibiotic therapy and improve clinical resolution of infections; however, further controlled in vivo efficacy and safety studies are warranted. From a clinical perspective, this prototype device provides a practical approach to UVC delivery, with a large beam diameter that allows treatment of the whole corneal surface in a single short exposure from an applicable working distance. Clinical trials and safety studies in companion animals are warranted to assess whether these antimicrobial effects can be demonstrated in vivo and to ensure UVC light therapy is safe.

## Author Contributions

**Oliver Joe Williams:** conceptualization, investigation, writing – original draft, methodology, writing – review and editing, project administration, data curation, validation, visualization. **Siân‐Marie Frosini:** conceptualization, methodology, investigation, writing – original draft, validation, visualization, writing – review and editing, supervision, data curation, resources. **Charlotte Dawson:** conceptualization, writing – review and editing, resources, supervision, methodology. **Maria‐Christine Fischer:** conceptualization, investigation, writing – review and editing, methodology, resources, supervision.

## Disclosure

*Artificial Intelligence Statement*: The authors have not used AI to generate any part of the manuscript.

## Ethics Statement

This study complies with the Guidelines for Ethical Research in Veterinary Ophthalmology (GERVO) and is exempt from approval by an ethics committee.

## Conflicts of Interest

The authors declare no conflicts of interest.

## Supporting information


**Table S1:** Antimicrobial susceptibilities of isolates included in the study. MDR was defined as resistance to three or more classes of antimicrobials.

## Data Availability

The data that support the findings of this study are available from the corresponding author upon reasonable request.

## References

[1] D. G. O'Neill , M. M. Lee , D. C. Brodbelt , D. B. Church , and R. F. Sanchez , “Corneal Ulcerative Disease in Dogs Under Primary Veterinary Care in England: Epidemiology and Clinical Management,” Canine Genetics and Epidemiology 4 (2017): 5. 28630713 10.1186/s40575-017-0045-5 PMC5471714

[2] E. M. James‐Jenks , C. L. Pinard , P. R. Charleboid , and G. Monteith , “Evaluation of Corneal Ulcer Type, Skull Conformation, and Other Risk Factors in Dogs: A Retrospective Study of 347 Cases,” Canadian Veterinary Journal 64, no. 3 (2023): 225–234. PMC9979749 36874547

[3] R. Thompson , “Lysozyme and the Antibacterial Properties of Tears,” Archives of Ophthalmology 25, no. 3 (1941): 491–509.

[4] S. Petersen‐Jones , “Quantification of Conjunctival Sac Bacteria in Normal Dogs and Those Suffering From Keratoconjunctivitis Sicca,” Veterinary and Comparative Ophthalmology 7, no. 1 (1997): 29–35.

[5] M.‐A. R. Salisbury , R. L. Kaswan , and J. Brown , “Microorganisms Isolated From the Corneal Surface Before and During Topical Cyclosporine Treatment in Dogs With Keratoconjunctivitis Sicca,” American Journal of Veterinary Research 56, no. 7 (1995): 880–884, 10.2460/ajvr.1995.56.07.880. 7574155

[6] J. M. Murphy , J. D. Lavach , and G. A. Severin , “Survey of Conjunctival Flora in Dogs With Clinical Signs of External Eye Disease,” Journal of the American Veterinary Medical Association 172, no. 1 (1978): 66–68. 624664

[7] M. R. Prado , M. F. G. Rocha , É. H. S. Brito , et al., “Survey of Bacterial Microorganisms in the Conjunctival Sac of Clinically Normal Dogs and Dogs With Ulcerative Keratitis in Fortaleza, Ceará, Brazil,” Veterinary Ophthalmology 8, no. 1 (2005): 33–37, 10.1111/j.1463-5224.2005.04061.x. 15644098

[8] L. Wang , Q. Pan , L. Zhang , Q. Xue , J. Cui , and C. Qi , “Investigation of Bacterial Microorganisms in the Conjunctival Sac of Clinically Normal Dogs and Dogs With Ulcerative Keratitis in Beijing, China,” Veterinary Ophthalmology 11, no. 3 (2008): 145–149, 10.1111/j.1463-5224.2008.00579.x. 18435654

[9] L. Wang , Q. Pan , Q. Xue , J. Cui , and C. Qi , “Evaluation of Matrix Metalloproteinase Concentrations in Precorneal Tear Film From Dogs With *Pseudomonas aeruginosa* –Associated Keratitis,” American Journal of Veterinary Research 69, no. 10 (2008): 1341–1345. 18828693 10.2460/ajvr.69.10.1341

[10] S. S. Twining , S. E. Kirschner , L. A. Mahnke , and D. Frank , “Effect of *Pseudomonas aeruginosa* Elastase, Alkaline Protease, and Exotoxin A on Corneal Proteinases and Proteins,” Investigative Ophthalmology and Visual Science 34, no. 9 (1993): 2699–2712. 8344792

[11] A. Suter , K. Voelter , S. Hartnack , B. M. Spiess , and S. A. Pot , “Septic Keratitis in Dogs, Cats, and Horses in Switzerland: Associated Bacteria and Antibiotic Susceptibility,” Veterinary Ophthalmology 21, no. 1 (2018): 66–75, 10.1111/vop.12480. 28557367

[12] R. Goss , V. J. Adams , C. Heinrich , et al., “Progressive Ulcerative Keratitis in Dogs in the United Kingdom: Microbial Isolates, Antimicrobial Sensitivity, and Resistance Patterns,” Veterinary Ophthalmology 27, no. 4 (2024): 330–346, 10.1111/vop.13160. 37933885

[13] M. R. Jinks , E. J. Miller , D. Diaz‐Campos , et al., “Using Minimum Inhibitory Concentration Values of Common Topical Antibiotics to Investigate Emerging Antibiotic Resistance: A Retrospective Study of 134 Dogs and 20 Horses With Ulcerative Keratitis,” Veterinary Ophthalmology 23, no. 5 (2020): 806–813. 32608547 10.1111/vop.12801

[14] E. Pedrotti , E. Bonacci , R. Kilian , et al., “The Role of Topical Povidone‐Iodine in the Management of Infectious Keratitis: A Pilot Study,” Journal of Clinical Medicine 11, no. 3 (2022): 848. 35160298 10.3390/jcm11030848 PMC8837158

[15] L. Spadea , E. Tonti , A. Spaterna , and A. Marchegiani , “Use of Ozone‐Based Eye Drops: A Series of Cases in Veterinary and Human Spontaneous Ocular Pathologies,” Case Reports in Ophthalmology 9, no. 2 (2018): 287–298, 10.1159/000488846. 29928225 PMC6006625

[16] T. P. Large , S. Mack , E. Villiers , and J. Oliver , “In Vitro Susceptibility of Canine Corneal Bacterial Pathogens to Three Cross‐Linking Protocols,” Veterinary Ophthalmology 26, no. S1 (2023): 134–142, 10.1111/vop.13006. 35713165

[17] H. T. Wolff , A. C. Piroth , H. Oltmanns , et al., “Commercially Available Antiseptics Show High In Vitro Efficacy Against Pathogens Most Commonly Associated With Canine and Feline Infectious Keratitis,” Frontiers in Veterinary Science 12 (2025): 1552230. 40470288 10.3389/fvets.2025.1552230 PMC12134761

[18] J. Jamieson , “The Influence of Light on the Development of Bacteria,” Nature 26, no. 663 (1882): 244–245, 10.1038/026244a0.

[19] R. P. Rastogi , n. Richa , A. Kumar , M. B. Tyagi , and R. P. Sinha , “Molecular Mechanisms of Ultraviolet Radiation‐Induced DNA Damage and Repair,” Journal of Nucleic Acids 2010, no. 1 (2010): 592980. 21209706 10.4061/2010/592980 PMC3010660

[20] N. G. Reed , “The History of Ultraviolet Germicidal Irradiation for Air Disinfection,” Public Health Reports 125, no. 1 (2010): 15–27. 10.1177/003335491012500105 PMC2789813 20402193

[21] C. S. Cockell and J. Knowland , “Ultraviolet Radiation Screening Compounds,” Biological Reviews 74, no. 3 (1999): 311–345. 10466253 10.1017/s0006323199005356

[22] C. Meulemans , “The Basic Principles of UV–Disinfection of Water,” Ozone: Science & Engineering 9 (1987): 299–313.

[23] M. Martinez‐Garcia , J. N. Sauceda‐Gálvez , I. Codina‐Torrella , M. M. Hernández‐Herrero , R. Gervilla , and A. X. Roig‐Sagués , “Evaluation of Continuous UVC Treatments and Its Combination With UHPH on Spores of *Bacillus subtilis* in Whole and Skim Milk,” Food 8, no. 11 (2019): 539. 10.3390/foods8110539 PMC6915690 31684085

[24] L. Eisenlöffel , T. Reutter , M. Horn , S. Schlegel , U. Truyen , and S. Speck , “Impact of UVC‐Sustained Recirculating Air Filtration on Airborne Bacteria and Dust in a Pig Facility,” PLoS One 14, no. 11 (2019): e0225047. 31697778 10.1371/journal.pone.0225047 PMC6837447

[25] M. Raeiszadeh and B. Adeli , “A Critical Review on Ultraviolet Disinfection Systems Against COVID‐19 Outbreak: Applicability, Validation, and Safety Considerations,” ACS Photonics 7, no. 11 (2020): 2941–2951, 10.1021/acsphotonics.0c01245. 37556269

[26] C. Song , R. Wen , J. Zhou , et al., “UV C Light From a Light‐Emitting Diode at 275 Nanometers Shortens Wound Healing Time in Bacterium‐ and Fungus‐Infected Skin in Mice,” Microbiology Spectrum 10, no. 6 (2022): e03424‐22, 10.1128/spectrum.03424-22. 36453911 PMC9769979

[27] T. P. Thai , D. H. Keast , K. E. Campbell , M. G. Woodbury , and P. E. Houghton , “Effect of Ultraviolet Light C on Bacterial Colonization in Chronic Wounds,” Ostomy/Wound Management 51, no. 10 (2005): 32–45. 16230765

[28] T. Dai , G. B. Kharkwal , J. Zhao , et al., “Ultraviolet‐C Light for Treatment of *Candida albicans* Burn Infection in Mice,” Photochemistry and Photobiology 87, no. 2 (2011): 342–349, 10.1111/j.1751-1097.2011.00886.x. 21208209 PMC3048910

[29] T. Dai , C. K. Murray , M. S. Vrahas , D. G. Baer , G. P. Tegos , and M. R. Hamblin , “Ultraviolet C Light for *Acinetobacter baumannii* Wound Infections in Mice: Potential Use for Battlefield Wound Decontamination?,” Journal of Trauma and Acute Care Surgery 73, no. 3 (2012): 661–667, 10.1097/TA.0b013e31825c149c. 22929495 PMC3463377

[30] S. Marasini , O. O. Mugisho , S. Swift , et al., “Effect of Therapeutic UVC on Corneal DNA: Safety Assessment for Potential Keratitis Treatment,” Ocular Surface 20 (2021): 130–138, 10.1016/j.jtos.2021.02.005. 33610742

[31] S. Marasini , S. J. Dean , S. Swift , et al., “Preclinical Confirmation of UVC Efficacy in Treating Infectious Keratitis,” Ocular Surface 25 (2022): 76–86. 35568373 10.1016/j.jtos.2022.05.004

[32] S. J. Dean , A. Petty , S. Swift , et al., “Efficacy and Safety Assessment of a Novel Ultraviolet C Device for Treating Corneal Bacterial Infections,” Clinical and Experimental Ophthalmology 39, no. 2 (2011): 156–163. 21105972 10.1111/j.1442-9071.2010.02471.x

[33] B. Turicea , D. K. Sahoo , R. A. Allbaugh , C. C. Stinman , and M. A. Kubai , “Novel Treatment of Infectious Keratitis in Canine Corneas Using Ultraviolet C (UV‐C) Light,” Veterinary Ophthalmology 28 (2025): 699–713, 10.1111/vop.13265. 39118265 PMC12274125

[34] M. Hoerdemann , D. K. Sahoo , R. A. Allbaugh , and M. A. Kubai , “Ultraviolet C (UV‐C) Light Therapy Inhibits Pathogens Associated With Equine Keratomycosis at Different Corneal Depths—An Ex Vivo Study,” Veterinary Ophthalmology 29, no. 2 (2026): e70110, 10.1111/vop.70110. 41399192 PMC12963518

[35] B. Turicea , D. K. Sahoo , R. A. Allbaugh , C. C. Stinman , and M. A. Kubai , “Antimicrobial Activity of Ultraviolet C Light as a Potential Novel Treatment for *Moraxella bovis* Infection—An In Vitro Study,” Veterinary Ophthalmology 29, no. 2 (2026): e70087, 10.1111/vop.70087. 41047748 PMC12963516

[36] M. Hoerdemann , D. K. Sahoo , R. A. Allbaugh , and M. A. Kubai , “Ultraviolet C (UV‐C) Light Therapy for Equine Ulcerative Keratomycosis—An In Vitro Study,” Veterinary Ophthalmology 29, no. 1 (2026): e70012, 10.1111/vop.70012.40045512

[37] A. J. McCarthy , E. M. Harrison , K. Stanczak‐Mrozek , et al., “Genomic Insights Into the Rapid Emergence and Evolution of MDR in *Staphylococcus pseudintermedius* ,” Journal of Antimicrobial Chemotherapy 70, no. 4 (2015): 997–1007. 25527273 10.1093/jac/dku496

[38] A.‐P. Magiorakos , A. Srinivasan , R. B. Carey , et al., “Multidrug‐Resistant, Extensively Drug‐Resistant and Pandrug‐Resistant Bacteria: An International Expert Proposal for Interim Standard Definitions for Acquired Resistance,” Clinical Microbiology and Infection 18, no. 3 (2012): 268–281. 21793988 10.1111/j.1469-0691.2011.03570.x

[39] A. C. Lorenzo‐Leal , W. Tam , A. Kheyrandish , M. Mohseni , and H. Bach , “Antimicrobial Activity of Filtered Far‐UVC Light (222 Nm) Against Different Pathogens,” BioMed Research International 2023 (2023): 2085140, 10.1155/2023/2085140. 37942030 PMC10630020

[40] S. Atta , C. Perera , S. Nayyar , R. P. Kowalski , and V. Jhanji , “An 18‐Year Overview of *Serratia marcescens* Ocular Infection,” Eye & Contact Lens 47, no. 8 (2021): 471–475, 10.1097/icl.0000000000000803. 34050088

[41] H. A. El‐Bialy and S. A. Abou El‐Nour , “Physical and Chemical Stress on Serratia Marcescens and Studies on Prodigiosin Pigment Production,” Annals of Microbiology 65, no. 1 (2015): 59–68, 10.1007/s13213-014-0837-8.

[42] M. Borić , T. Danevčič , and D. Stopar , “Prodigiosin From Vibrio sp. DSM 14379; A New UV‐Protective Pigment,” Microbial Ecology 62, no. 3 (2011): 528–536, 10.1007/s00248-011-9857-0. 21547449

[43] W. E. Feldman , “Concentrations of Bacteria in Cerebrospinal Fluid of Patients With Bacterial Meningitis,” Journal of Pediatrics 88, no. 4 (1976): 549–552. 3635 10.1016/s0022-3476(76)80003-0

[44] S. Kaidzu , K. Sugihara , M. Sasaki , et al., “Safety Evaluation of Far‐UV‐C Irradiation to Epithelial Basal Cells in the Corneal Limbus,” Photochemistry and Photobiology 99, no. 4 (2023): 1142–1148, 10.1111/php.13750. 36437576

[45] S. Marasini , S. Dean , and J. P. Craig , “Safety of Therapeutic Ultraviolet C Light Application at the Limbus,” Investigative Ophthalmology & Visual Science 65, no. 7 (2024): 4121.

[46] D. Ramírez‐Gamboa , A. L. Díaz‐Zamorano , E. R. Meléndez‐Sánchez , et al., “Photolyase Production and Current Applications: A Review,” Molecules 27, no. 18 (2022): 5998, 10.3390/molecules27185998. 36144740 PMC9505440

[47] M. Zhang , L. Wang , and D. Zhong , “Photolyase: Dynamics and Electron‐Transfer Mechanisms of DNA Repair,” Archives of Biochemistry and Biophysics 632 (2017): 158–174, 10.1016/j.abb.2017.08.007. 28802828 PMC5650541

[48] C. T. Lin and S. M. Petersen‐Jones , “Antibiotic Susceptibility of Bacterial Isolates From Corneal Ulcers of Dogs in Taiwan,” Journal of Small Animal Practice 48, no. 5 (2007): 271–274, 10.1111/j.1748-5827.2007.00348.x. 17425695

[49] K. E. Hindley , A. D. Groth , M. King , K. Graham , and F. M. Billson , “Bacterial Isolates, Antimicrobial Susceptibility, and Clinical Characteristics of Bacterial Keratitis in Dogs Presenting to Referral Practice in Australia,” Veterinary Ophthalmology 19, no. 5 (2016): 418–426. 26522379 10.1111/vop.12325

[50] A. Tsvetanova , R. M. Powell , K. A. Tsvetanov , K. M. Smith , and D. J. Gould , “Melting Corneal Ulcers (Keratomalacia) in Dogs: A 5‐Year Clinical and Microbiological Study (2014–2018),” Veterinary Ophthalmology 24, no. 3 (2021): 265–278. 33794048 10.1111/vop.12885

